# How often do physicians review medication charts on ward rounds?

**DOI:** 10.1186/1472-6904-8-9

**Published:** 2008-09-29

**Authors:** Khang Li Looi, Peter N Black

**Affiliations:** 1 Dept of Cardiology, Auckland City Hospital, Private Bag 92024, Auckland 1142, New Zealand; 2 Dept of Pharmacology & Clinical Pharmacology, University of Auckland, Private Bag 92019, Auckland 1142, New Zealand

## Abstract

**Background:**

Prescribing errors are common in hospital settings. Regular review of medication charts is recommended as a way to reduce errors but it is not clear how often this happens. The aim of this study was to determine the frequency with which specialist physicians reviewed medication charts during ward rounds.

**Methods:**

An observer noted how often consultant physicians at Auckland City Hospital reviewed medication charts during ward rounds. The physicians were not aware that they were being observed.

**Results:**

Twenty-one physicians were observed over a 26 week period. The general physicians reviewed the medication charts on 77% of occasions (range: 45% – 100%) during routine ward rounds and 65% of the time (range: 41% – 80%) on post admission rounds. Subspecialty physicians who did not see more than 8 patients on their rounds reviewed medication charts more frequently (88%) than those specialties where more than 8 patients were seen on average (61%).

**Conclusion:**

The physicians did not review medication charts on all ward rounds and there was considerable variation in how often they did this. There is some evidence that the frequency with which charts are reviewed decreases as the number of patients seen increases. More efforts should be made to encourage regular review of medication charts.

## Background

Prescribing errors are common in the hospital. In different studies adverse drug events have been reported to occur in 0.7% to 6.5% of hospital admissions [1]. 28% to 56% of these adverse drug events were thought to be preventable [2]. Bates et al estimated that each preventable adverse event leads to an additional cost of US $4,685 and increase in the length of stay of 4.6 days [3]. A number of interventions have been proposed to reduce medication errors including regular review of medication charts by doctors on ward rounds [2]. A case can be made for consultant staff to review all of the medication charts on every ward round that they go on. There is evidence that consultants make fewer prescribing errors than their junior medical staff [4,5] and they are well placed to detect errors made by the junior medical staff. If they review medication charts on each ward round it also emphasizes the importance of doing this to the junior medical staff. However we are not aware of any previous studies that document how often consultants review medication charts on ward rounds.

## Methods

We determined the frequency with which consultant physicians at Auckland City Hospital (ACH) (Auckland, New Zealand) reviewed medication charts during their ward rounds. The study was undertaken by a medical relief registrar (KLL) who was assigned to cover other registrars who were on leave. From 26^th ^June 2006 till 10^th ^December 2006 (26 weeks) she rotated through general medicine and a number of subspecialties. Most attachments were for 1–2 weeks. At ACH, most of the patients presenting with acute medical problems are admitted to the General Medical Service with only a minority presenting directly to the medical subspecialties. A proportion of these patients will be subsequently transferred to a subspecialty although the majority remain under the care of General Medicine. Patients admitted to the General Medical Service are seen by a consultant physician on a post admission round within 24 hours of arriving in hospital. On routine ward rounds the patients have been in hospital for more than 24 hours and have been seen by a consultant physician at least once before. The medication charts at ACH are completed manually by the junior medical staff when the patient is admitted. Staff pharmacists are not present on the ward rounds. Although they see most of the medication charts of patients admitted to hospital they only talk to the patient and take a medication history on about 10% of occasions. On consultant ward rounds KLL recorded how often the consultant reviewed the medication chart i.e. how often the consultant looked at the medication chart and where appropriate suggested changes. The consultant needed to scrutinise the medication chart for 15 seconds or more before they were recorded as having reviewed the medication chart. None of the consultants were aware of the study. This was not felt to pose a problem because this was an audit and did not involve any intervention or any change to routine practice. Because this was an audit and did not involve an intervention we were not required to seek Ethics Committee approval. The consultants were not prompted to review the medication chart by the registrar. The study was carried out for a period of 26 weeks.

The results were expressed as mean ± SD and range. Comparisons were performed using an unpaired Student's t-test.

## Results

Twenty-one consultants were observed during the course of the study. Eight were general physicians. The remainder were from gastroenterology (3), the liver transplant service (5), haematology (2), cardiology (2) and renal medicine (1). In general medicine each consultant was observed twice i.e. on a routine ward round and on a post-admission round. The subspecialty physicians were observed on either one or two occasions. All the patients cared for by these services were over the age of 16 years.

In general medicine the frequency of with which the physicians reviewed medication charts on routine ward rounds (Table 1) ranged from 45 to 100% (mean = 77 ± 16%). Between 6 and 15 patients were seen on these wards rounds. In contrast on the post admission rounds between 11 and 21 patients were seen. The medication charts were reviewed less frequently on the post admission rounds (range = 41 – 80%, mean = 65 ± 14%) although this difference was not statistically significant (p = 0.14).

**Table 1:** Frequency of review of medication charts on general medicine rounds

	**No. of Patients Seen on Ward Round**	**Frequency of Medication Chart Review**
**Routine Ward Rounds**		
A	7	100%
B	15	87%
C	8	87%
D	11	82%
E	6	67%
F	8	75%
G	11	73%
H	11	45%
Mean	10	77%
		
**Post Admission Rounds**		
A	15	80%
B	12	67%
C	17	41%
D	17	76%
E	11	64%
F	17	76%
G	12	50%
H	21	67%
Mean	15	65%

Each letter (from A to H) represents an individual physician. Each physician was observed on both a routine ward round and on a post admission round.

The average number of patients seen in the haematology and liver transplant services (Table 2) was five with no more than 8 patients seen on any round. On these services the medication charts were reviewed more frequently (range 75% – 100%, mean = 88 ± 12%). In the other subspecialties (Table 2) more patients were seen (mean = 9, range = 4 – 16) and the medication charts were reviewed less frequently (mean = 61 ± 18%, p = 0.003). On most ward rounds only one consultant was present. The exception was the liver transplant service where several consultants took part in each round but only one took responsibility for reviewing the medication chart.

**Table 2:** Frequency of review of medication charts on subspecialty ward rounds

**Specialty**	**No. of Patients Seen on Ward Round**	**Frequency of Medication Chart Review**
**Haematology**		
A	6	83%
	3	100%
B	3	100%
	8	75%
Mean	5	90%
		
**Liver Transplant**		
A	5	100%
B	5	100%
C	4	75%
D	5	80%
E	5	80%
Mean	5	87%
		
**Gastroenterology**		
A	11	45%
B	7	43%
	5	80%
C	10	50%
	9	44%
Mean	8	52%
		
**Cardiology**		
A	13	61%
B	4	75%
Mean	8	68%
		
**Renal Medicine**		
A	16	87%

In each specialty each of the physicians who were observed is represented by a separate letter. Each physician was observed on either one or two occasions.

## Discussion

We found that a significant proportion of medication charts were not reviewed by physicians on their ward rounds. We are not aware of other studies that have looked at this. This may reflect the difficulty of conducting this type of study. If physicians know that they are being observed their behaviour may change. McHugh found that when doctors were informed that their prescribing was being audited the number of errors decreased significantly [4]. Our study was only possible because there are medical registrars in our hospital who provide leave cover and who work on a different team every one or two weeks. This provided the opportunity to observe a range of physicians without them being aware of the study.

A number of strategies have been proposed to reduce the frequency of prescribing errors including computerised physician order entry and pharmacist participation in ward rounds. The presence of a pharmacist on a ward round is associated with a lower rate of prescribing errors [6]. The review of medication charts by pharmacists demonstrates the value of having another individual check for errors. At ACH pharmacists do not usually participate in ward rounds. However consultant physicians are well placed to detect errors. Senior physicians are more experienced that junior medical staff and a number of studies have found that they make fewer errors when they prescribe medicines [4,5]. We believe that if consultants review most, if not all, of the medication charts on ward rounds it will increase the chance of detecting errors. We are not aware of any studies that examine how often consultant physicians detect medication errors on ward rounds but our experience is that this occurs not infrequently.

At the time of writing computerised prescribing has not been implemented at Auckland City Hospital. Numerous studies have shown that computerised prescribing can reduce medication errors [7,8]. However, these systems still have some shortcomings [8] and they do not supplant the need for physicians to review the medication charts closely.

The charts were more likely to be reviewed in subspecialties with eight or fewer patients. This is not altogether surprising. As the workload increases the time available to review the medication chart will decrease. This may explain why medication charts were reviewed less often in General Medicine on the post admitting rounds when more patients were seen than on routine ward rounds. This difference was not statistically significant but may well have been if the study had been larger. Prescribing chemotherapy for cancer patients and immunosuppressive medication for transplant patients can be complex and there is the risk of significant toxicity. This could also explain why the consultants in haematology and on the liver transplant service were careful to examine the medication charts on most rounds.

This study has some limitations. Only twenty-one physicians were studied and each physician was only observed on one or two occasions. The need to conduct the study without the physicians being aware that they were observed limited our ability to do a larger study. The study was only conducted in a single hospital and we are not certain that the findings can be generalised to other hospitals but we suspect that this is the case. When Davis et al studied the frequency and type of adverse events in New Zealand hospitals their findings paralleled those from Australia and the United Kingdom [9]. Another limitation is that we did not count the number of prescription errors on the medication charts and we do not know if there was a relation between the frequency with which charts were reviewed and the number of errors. It would be of interest to study this although anyone doing this would face the same challenges that we did in conducting a study without physicians being aware that they were observed.

## Conclusion

In summary we found that physicians only reviewed medication charts about two thirds of the time and the frequency decreased as the number of patients increased. Although there is no direct evidence that consultant review of medication charts reduces prescribing errors there is good reason to believe that this is the case and we would advocate reviewing all the medication charts on every ward round.

## Competing interests

The authors declare that they have no competing interests.

## Authors' contributions

PNB conceived the idea for the study. PNB and KLL were jointly responsible for the study design. KLL carried out the study. Both authors shared responsibility for analysing the data and writing the manuscript and both authors have approved the final manuscript.

## Pre-publication history

The pre-publication history for this paper can be accessed here:


